# How to identify cell material in a single ice grain emitted from Enceladus or Europa

**DOI:** 10.1126/sciadv.adl0849

**Published:** 2024-03-22

**Authors:** Fabian Klenner, Janine Bönigk, Maryse Napoleoni, Jon Hillier, Nozair Khawaja, Karen Olsson-Francis, Morgan L. Cable, Michael J. Malaska, Sascha Kempf, Bernd Abel, Frank Postberg

**Affiliations:** ^1^ Department of Earth and Space Sciences, University of Washington, Seattle, WA, USA.; ^2^ Institute of Geological Sciences, Freie Universität Berlin, Berlin, Germany.; ^3^ Faculty of Science, Technology, Engineering and Mathematics, The Open University, Milton Keynes, UK.; ^4^ Jet Propulsion Laboratory, California Institute of Technology, Pasadena, CA, USA.; ^5^ Laboratory for Atmospheric and Space Physics, University of Colorado, Boulder, CO, USA.; ^6^ Institute of Chemical Technology, University of Leipzig, Leipzig, Germany.; ^7^ Leibniz-Institute of Surface Engineering (IOM), Leipzig, Germany.

## Abstract

Icy moons like Enceladus, and perhaps Europa, emit material sourced from their subsurface oceans into space via plumes of ice grains and gas. Both moons are prime targets for astrobiology investigations. Cassini measurements revealed a large compositional diversity of emitted ice grains with only 1 to 4% of Enceladus’s plume ice grains containing organic material in high concentrations. Here, we report experiments simulating mass spectra of ice grains containing one bacterial cell, or fractions thereof, as encountered by advanced instruments on board future space missions to Enceladus or Europa, such as the SUrface Dust Analyzer onboard NASA’s upcoming Europa Clipper mission at flyby speeds of 4 to 6 kilometers per second. Mass spectral signals characteristic of the bacteria are shown to be clearly identifiable by future missions, even if an ice grain contains much less than one cell. Our results demonstrate the advantage of analyses of individual ice grains compared to a diluted bulk sample in a heterogeneous plume.

## Teaser

Future space missions can identify even a tiny fraction of a cell, if present in an ice grain emitted from an ocean world.

## INTRODUCTION

The reliable identification and quantification of biosignatures on extraterrestrial ocean worlds are key to the search for life in our Solar System (*1*, *2*). Saturn’s moon Enceladus and, potentially, Jupiter’s moon Europa emit plumes of gas and ice grains sourced from subsurface water into space (*3*, *4*). For Enceladus, evidence from multiple Cassini measurements indicates that the plume is sourced from its liquid water ocean and not a near-surface reservoir (*5*). The compositions of single ice grains can be sampled in situ during spacecraft flybys by impact ionization mass spectrometers, such as the Cosmic Dust Analyzer (CDA) (*6*) onboard the past Cassini mission, the SUrface Dust Analyzer (SUDA) (*7*) onboard NASA’s upcoming Europa Clipper mission (*8*), or even more capable instruments proposed for future Enceladus missions, such as the ENceladus Ice Analyzer (ENIA) (*9*, *10*) or the High Ice Flux Instrument (HIFI) (*11*). Other capable impact ionization mass spectrometers include the Interstellar Dust Experiment (IDEX) onboard NASA’s upcoming Interstellar Mapping and Acceleration Probe (IMAP) (*12*) and the Destiny^+^ Dust Analyzer onboard JAXA’s upcoming DESTINY^+^ mission (*13*).

Analysis of CDA data collected in the Saturnian System revealed that Enceladus’s subsurface ocean interacts hydrothermally with the moon’s rocky core (*14*). The ocean is salty (*15*), similar in salinity to Earth’s oceans; it also contains a diverse complement of organic material, including low-mass volatile, nitrogen- and oxygen-bearing compounds (*16*), and complex, refractory macromolecules (*17*). While most of the Enceladus plume grains contain only traces of salts and organics, others show either inorganic or organic compounds in markedly enhanced concentrations (*15*, *17*, *18*). The moon’s astrobiological relevance recently further increased due to the detection of hydrogen cyanide (*19*) and orthophosphates (*20*) in the emitted plume material. Hydrogen cyanide is an important precursor in the synthesis of nucleobases and amino acids (*21*, *22*). The detection of phosphates showed that phosphorus is an abundant element in Enceladus’s ocean, in quantities that could possibly support the origin, maintenance, or growth of microbial life.

Several mission concepts and techniques have been proposed to look for prebiotic chemistry or even evidence of life on Enceladus or Europa (*8*, *10*, *11*, *23*–*28*). Of the techniques discussed, to our knowledge, only impact ionization mass spectrometry can analyze the unique compositions of individual plume ice grains, typically only a few micrometers in diameter. The key strength of analyzing ice grains individually—in contrast to collecting several billions of them and then analyzing the integrated composition—is to take advantage of the strong chemical partitioning of different compounds into different ice grains that appear to be characteristic for Enceladus and possibly also for Europa.

On Earth, the ocean is covered by a surface microlayer that covers approximately 70% of the planet’s surface (*29*). The microlayer consists of a gelatinous biofilm, hosting a distinct microbial community that is three to five orders of magnitude higher in density than in the bulk water phase (*30*, *31*). After lofting by, for example, bursting bubbles (*32*, *33*), organics, and cells from this layer can initiate ice crystal formation in clouds (*34*). On Enceladus, bacterial cells or fragments thereof, if present, would thus be likely accumulated in an organic microlayer on top of the oceanic surface, as hypothesized by Porco *et al*. (*35*). The macromolecular, refractory organics detected in Enceladan ice grains are thought to form from such a microlayer (*17*). These organics occur in only a few percent of plume ice grains from Enceladus; therefore, if cell material is present, it would probably be incorporated into only very few individual grains, but with a relatively high concentration within each of these grains. Bacterial cells from an oceanic surface microlayer could be incorporated into ice grains due to the bursting of gas bubbles ascending through Enceladus’s ocean (*17*, *35*) or controlled boiling (*36*). Potential cell densities in Enceladus’s ocean are only loosely constrained to 5 × 10^−6^ to 5 × 10^3^ cells/ml (*5*, *37*), and estimated cell densities in the average Enceladus plume material increased to 1 × 10^3^ to 8.5 × 10^7^ cells/ml due to concentration mechanisms (*27*, *35*, *38*).

While CDA was able to analyze the compositions between 30 and 300 ice grains during a single passage through Enceladus’s plume, future instruments, such as SUDA (*7*), ENIA (*10*), or HIFI (*11*), would be able to sample 10,000 to 100,000 individual ice grains in a diameter range from 0.5 to 50 μm per plume flythrough. The total ice grain emission rate of Enceladus’s plume is 15 to 65 kg/s, of which ~10% escape the moon’s gravity and enter Saturn’s E ring (*39*, *40*).

To simulate a potential scenario for such instruments, in which cell material is present in only a small number of emitted grains, but with a relatively high concentration therein, we conducted laboratory analog experiments using the Laser Induced Liquid Beam Ion Desorption (LILBID) approach (*41*) with untreated cell material. The LILBID approach has been used previously to successfully predict the mass spectral signatures and detection limits of various organic compounds in ice grains (*42*–*45*) including those of potential biosignatures in mass spectra of emitted ice grains, namely, amino acids, fatty acids, and peptides (*46*, *47*), as well as DNA, lipids, and metabolites extracted from *Escherichia coli* and *Sphingopyxis alaskensis* cultures (*48*). The results of these experiments demonstrate that the investigated molecules will produce characteristic signals in mass spectra of ice grains, even if the molecules are present in concentrations at the parts per million or parts per billion level. According to these experiments (*46*–*48*), and supported by other experimental (*49*) and modeling studies (*50*, *51*), relative velocities of 4 to 6 km/s appear to be an optimal speed window for biosignature detection using mass spectrometry via spacecraft-ice grain encounters. However, to date, biosignatures have not been identified on extraterrestrial ocean worlds with this or any other method.

## RESULTS

Here, we present results from LILBID experiments with the same *S. alaskensis* culture studied by Dannenmann *et al.* (*48*). *S. alaskensis* is an ultrasmall (volume < 0.1 μm^3^) bacterium, extracted from various cold marine environments (*52*), and potentially capable of fitting into emitted micrometer-sized ice grains. While these bacteria only require low nutrient fluxes for survival and growth, they can use molecular hydrogen as an energy source (*53*), a compound that is abundant in Enceladus’s plume (*54*) and therefore may serve as a good example organism for putative life in the Enceladan ocean. In contrast to previous work, we do not use cell extracts, and we simulate an even more realistic case by using the complete, untreated cell material. We simulate the case of a 15-μm-diameter ice grain, formed around a nucleation core of one single inactivated bacterial cell or small fragments thereof, emitted by an ocean world plume and encountered by a SUDA-type detector during a spacecraft flyby at 4 to 6 km/s (*39*). This constitutes a worst case for Enceladus, where ice grains are typically much smaller (1 to 5 μm in diameter) (*39*, *40*) and the concentration of cell materials would be higher relative to the total grain volume. Cell density (in cells per milliliter) calculations can be found in the “Preparation of *S. alaskensis* cell samples” section in Materials and Methods. In our LILBID experiments, the *S. alaskensis* samples were vertically injected into a vacuum within a 15-μm-diameter water beam that disintegrates into droplets after typically 2 to 3 mm. Pulsed infrared laser radiation (wavelength of 2840 nm) desorbs cations and anions from these individual droplets (thereby simulating the impact ionization process; see the “Preparation of *S. alaskensis* cell samples” and “Simulating mass spectra of single ice grains using the laboratory Laser Induced Liquid Beam Ion Desorption (LILBID) facility” sections in Material and Methods), which are in turn detected with a time-of-flight (TOF) mass spectrometer (*41*, *55*).

### Cationic mass spectra

**Fig. 1.**
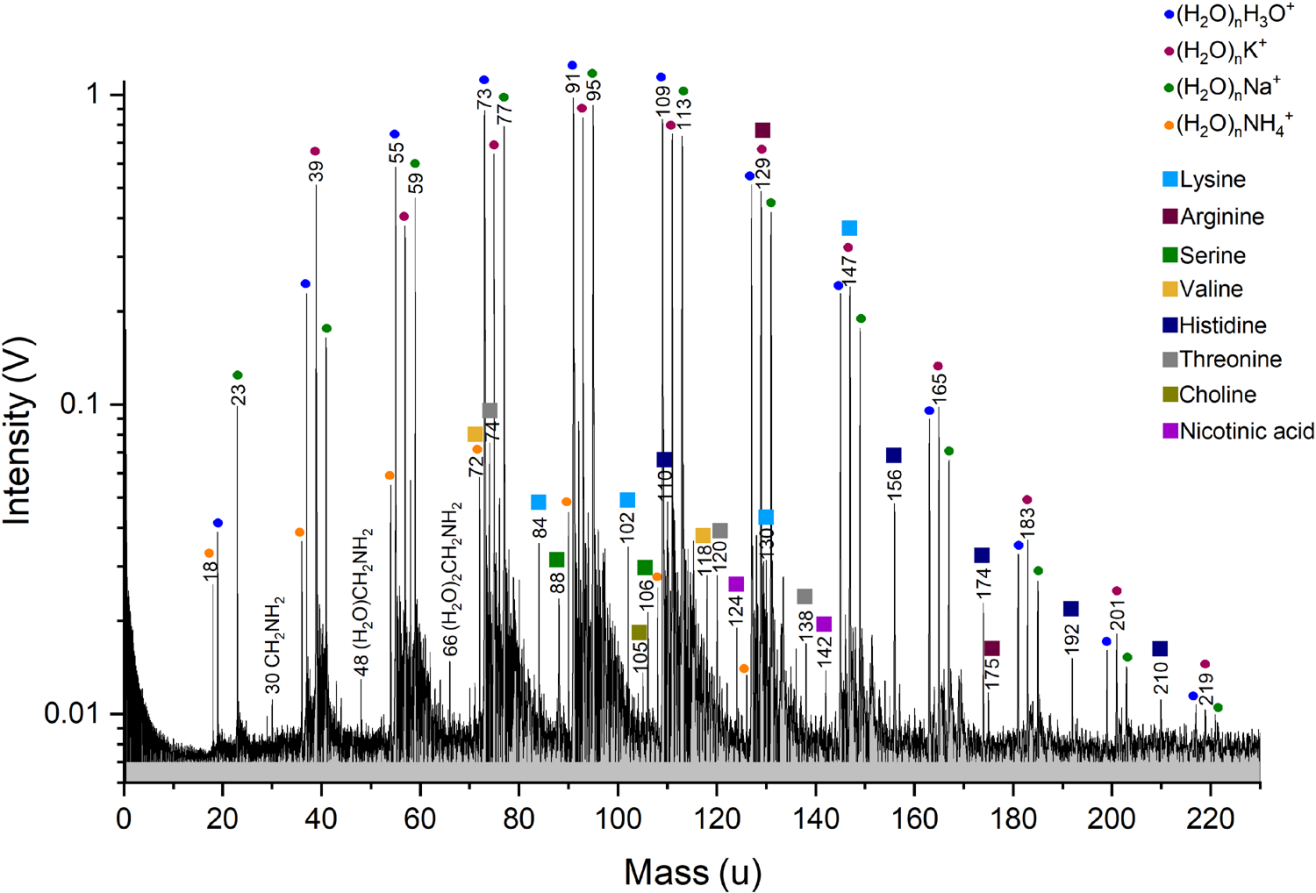
Baseline corrected cationic mass spectrum of the cell material equivalent to one *S. alaskensis* cell in a 15-μm-diameter H_2_O droplet. Although the mass spectrum is dominated by water, sodium-water, potassium-water, and ammonium-water clusters, amino acids together with other metabolic intermediates from the *S. alaskensis* cell can be identified. The spectrum is an average of 224 individual spectra recorded with instrument settings corresponding to ice grain impact speeds onto spaceborne detectors of 4 to 6 km/s (*41*).

Figure 1 shows a cationic mass spectrum that simulates the case of one bacterial cell in a single 15-μm ice grain emitted via an ocean world plume and detected by a spaceborne mass spectrometer. The mass spectrum is dominated by water (H_2_O)_n_H_3_O^+^, potassium-water (H_2_O)_n_K^+^, sodium-water (H_2_O)_n_Na^+^, and ammonium-water (H_2_O)_n_NH_4_^+^ clusters from the water matrix and likely the cell’s cytosol. The spectrum exhibits peaks due to protonated amino acids and their fragments, either metabolic intermediates or fragments of the bacterium’s proteins. Amino acid peak amplitudes vary due to differing cellular concentrations of these species, as well as sensitivity variations of the ionization method to different amino acids at a given concentration. The lowest detection limits in cation mode are found for amino acids having basic side chains (*46*).

All identified cationic organic species are summarized in Table 1. Although the molecular peak of lysine interferes with a potassium-water cluster [H_2_O]_6_K^+^ at mass/charge ratio (*m/z*) 147, fragments of lysine can still be detected at *m/z* 84, 102, and 130. The fragment at *m/z* 84 clusters with water and is represented by a peak at *m/z* 102. Protonated arginine molecules are detected at *m/z* 175. An arginine fragment interferes with a potassium-water cluster [H_2_O]_5_K^+^ at *m/z* 129. Protonated serine is identified at *m/z* 106, together with a serine fragment at *m/z* 88. Peaks at *m/z* 120 and 74 can be assigned to protonated threonine and a threonine fragment. The identified fragments of lysine, arginine, serine, and threonine, respectively, agree with previous LILBID experiments using these amino acids in H_2_O (*46*). Peaks at *m/z* 118 and 72 can be assigned to the protonated valine molecule and a valine fragment. We note, however, that the valine fragment interferes with an ammonium-water cluster [H_2_O]_3_NH_4_^+^. Protonated histidine is identified at *m/z* 156, together with a histidine fragment at *m/z* 110. This fragment has also been observed from histidine in other analog experiments for space-based ice grain mass spectrometry (*56*). [CH_2_NH_2_]^+^ at *m/z* 30 is a typical fragment of amines and has previously been observed in LILBID experiments with amino acids (*46*) and extracts from *S. alaskensis* (*48*). Two more protonated metabolic intermediates, choline and nicotinic acid, are identified at *m/z* 105 and 124.

**Table 1.** Organic species identified in cationic LILBID mass spectra of *S. alaskensis* cells (Fig. 1). Water clusters of organic species are not listed. SNR, signal-to-noise ratio; *m/z*, mass/charge ratio.

*m/z*	SNR	Identified species	Molecular formula	Description
30	2	Methaniminium cation	[CH_2_NH_2_]^+^	Unspecific organic fragment
72	8^*^	Valine-COOH^−^	[C_4_H_10_N]^+^	Amino acid fragment
74	10	Threonine-COOH^−^	[C_3_H_8_NO]^+^	Amino acid fragment
84	5	Lysine-COOH^−^-NH_3_	[C_5_H_10_N]^+^	Amino acid fragment
88	3	Serine-OH^−^	[C_3_H_6_NO_2_]^+^	Amino acid fragment
105	2	Protonated choline molecule	[C_5_H_14_NO]H^+^	Metabolic intermediate
106	3	Protonated serine molecule	[C_3_H_7_NO_3_]H^+^	Amino acid parent
110	7	Histidine-COOH^−^	[C_5_H_9_N_3_]^+^	Amino acid fragment
118	4	Protonated valine molecule	[C_5_H_11_NO_2_]H^+^	Amino acid parent
120	4	Protonated threonine molecule	[C_4_H_9_NO_3_]H^+^	Amino acid parent
124	3	Protonated nicotinic acid molecule	[C_6_H_5_NO_2_]^+^	Metabolic intermediate
129	63^*^	Arginine-COOH^−^	[C_5_H_14_N_4_]^+^	Amino acid fragment
130	4	Lysine-NH_2_^−^	[C_6_H_12_NO_2_]^+^	Amino acid fragment
147	30^*^	Protonated lysine molecule	[C_6_H_14_N_2_O_2_]H^+^	Amino acid parent
156	7	Protonated histidine molecule	[C_6_H_9_N_3_O_2_]H^+^	Amino acid parent
175	2	Protonated arginine molecule	[C_6_H_14_N_4_O_2_]H^+^	Amino acid parent

*Potential interference observed.

We conducted further LILBID experiments with lower cell densities to determine the detection limits of mass spectral signatures of *S. alaskensis*. These results show that the amplitudes of peaks characteristic of *S. alaskensis* decrease with decreasing cell density. Two non-interfering *S. alaskensis*–related peaks with signal-to-noise ratios (SNRs) ≥7 in Fig. 1, namely, *m/z* 74 and 110, are found to be detectable down to 50 times lower cell densities (fig. S1) than needed to simulate the case of a single 15-μm ice grain that is formed around a nucleation core of a bacterial cell.

### Anionic mass spectra

**Fig. 2.**
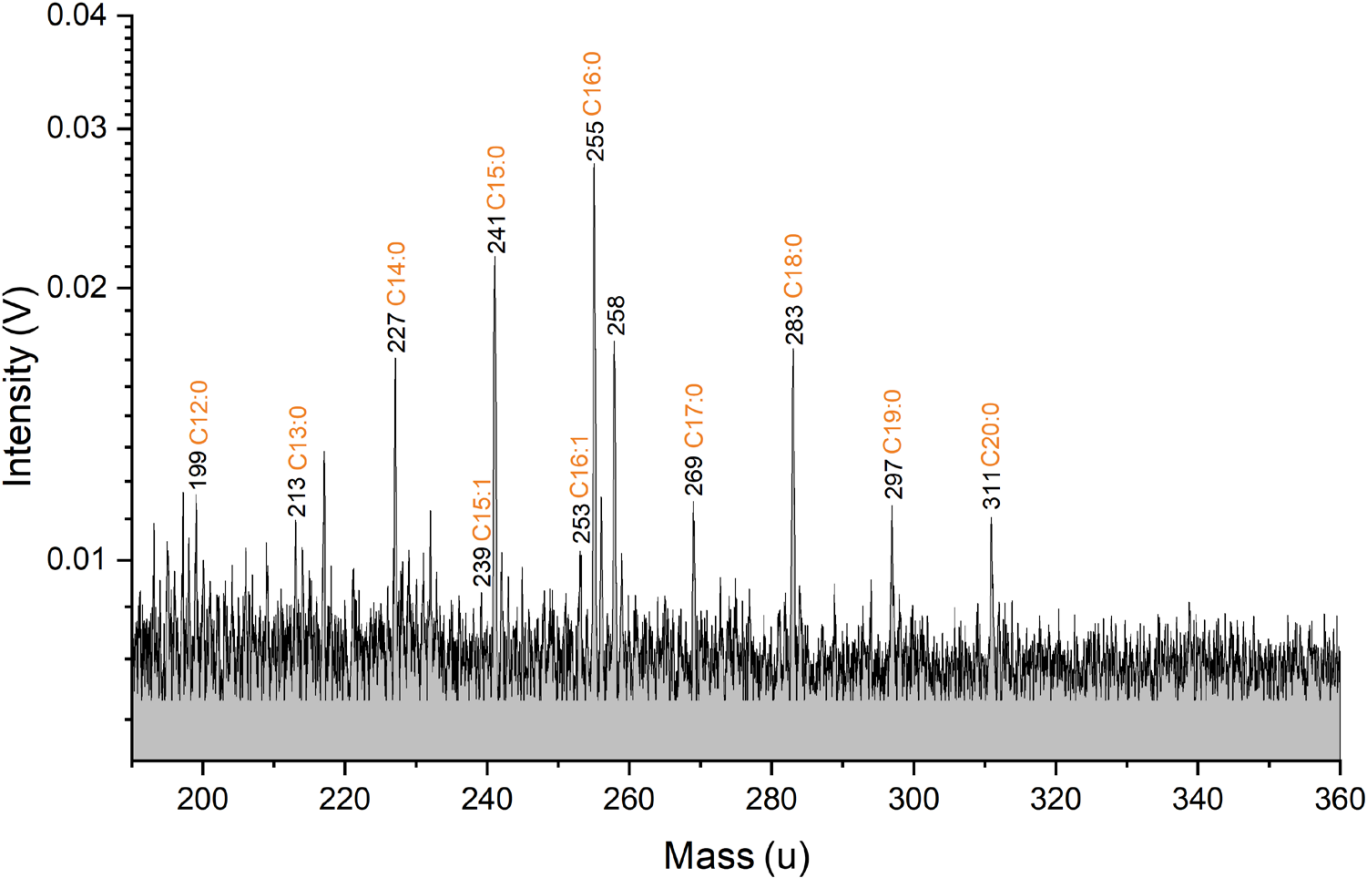
Section (*m/z* 190 to 360) of a baseline corrected anionic mass spectrum of the cell material equivalent to one *S. alaskensis* cell in a 15-μm-diameter H_2_O:isopropanol (1:1 vol:vol) droplet. The full spectrum is shown in fig. S2. Deprotonated molecules of saturated and unsaturated fatty acids with 12 to 20 carbon numbers can be identified, with C15:0, C16:0, and C18:0 having the highest amplitudes. The spectrum is an average of 339 individual spectra recorded with instrument settings corresponding to ice grain impact speeds of 4 to 6 km/s onto a spaceborne detector (*41*). A control anionic spectrum of H_2_O:isopropanol (1:1 vol:vol) without *S. alaskensis* cells is shown in fig. S3.

Figure 2 shows an anionic mass spectrum that simulates the case of one bacterial cell in a single 15-μm ice grain emitted in an ocean world plume and detected by a spaceborne mass spectrometer. The full spectrum is shown in fig. S2. In contrast to the cation mode, in which we found polar cell constituents, the anion mode is particularly sensitive to nonpolar cell constituents, such as lipids and their fragments (*48*). Because of the lipids’ poor water solubilities, we measured the *S. alaskensis* cells in an H_2_O-isopropanol (1:1 vol:vol) matrix. With the applied instrument settings, the matrix (without cells) only produced a few peaks at *m/z* > 190, easily distinguishable from the peaks derived from the lipids (fig. S3).

In the measured sample (Fig. 2), sequences of deprotonated fatty acids (unbranched saturated and unsaturated) with 12 to 20 carbon atoms are identifiable at *m/z* > 190, representing fragments of the bacterial lipids. Tetradecanoic acid (C14:0), pentadecanoic acid (C15:0), hexadecanoic acid (C16:0), and octadecanoic acid (C18:0) produce the strongest peaks from *S. alaskensis* cells. The fatty acid pattern observed in our experiments matches the fatty acid pattern of lipids extracted from *S. alaskensis* and analyzed using LILBID (*48*). Identified lipid fragments are summarized in Table 2.

**Table 2.** Deprotonated fatty acids, i.e., fragments of the membrane lipids, identified in anionic LILBID mass spectra of *S. alaskensis* cells (Fig. 2).

*m/z*	SNR	Identified species	Molecular formula
199	2	Dodecanoic acid (C12:0)	[CH_3_(CH_2_)_10_COO]^−^
213	2	Tridecanoic acid (C13:0)	[CH_3_(CH_2_)_11_COO]^−^
227	3	Tetradecanoic acid (C14:0)	[CH_3_(CH_2_)_12_COO]^−^
239	2	10-cis-Pentadecenoic acid (C15:1)	[CH_3_(CH_2_)_3_CH=CH(CH_2_)_8_COO]^−^
241	3	Pentadecanoic acid (C15:0)	[CH_3_(CH_2_)_13_COO]^−^
253	2	9-cis-Hexadecenoic acid (C16:1)	[CH_3_(CH_2_)_5_CH=CH(CH_2_)_7_COO]^−^
255	4	Hexadecanoic acid (C16:0)	[CH_3_(CH_2_)_14_COO]^−^
269	2	Heptadecanoic acid (C17:0)	[CH_3_(CH_2_)_15_COO]^−^
283	3	Octadecanoic acid (C18:0)	[CH_3_(CH_2_)_16_COO]^−^
297	2	Nonadecanoic acid (C19:0)	[CH_3_(CH_2_)_17_COO]^−^
311	2	Eicosanoic acid (C20:0)	[CH_3_(CH_2_)_18_COO]^−^

As expected, the amplitudes of the *S. alaskensis* lipid fragment peaks, i.e., fatty acids, decrease with decreasing cell density. Having SNR ≥3 in Fig. 2, pentadecanoic acid (C15:0), hexadecanoic acid (C16:0), and octadecanoic acid (C18:0) are still detectable at cell densities 100 times lower (fig. S4) than needed to simulate the case of a single 15-μm ice grain that is formed around a nucleation core of a single bacterial cell.

## DISCUSSION

The clear detectability of signatures from *S. alaskensis* in both polarity LILBID spectra demonstrates that signatures of bacteria potentially embedded in ice grains and emitted via an ocean world plume would be readily detectable using impact ionization mass spectrometry. Our experiments show that even if only 1% of a cell’s constituents are contained in a 15-μm ice grain (or one cell in a 70-μm-diameter grain), the bacterial signatures would be apparent in the spectral data. We simulated a very low cell density for emitted ice grains. Cell densities in a typical ice grain of the most common size regime of 1 to 5 μm (*39*, *40*) would be approximately 100 times higher than in our experiment (see the “Preparation of *S. alaskensis* cell samples” section in Materials and Methods). Thus, the characteristic signatures arising from approximately 0.01% of the constituents of a single cell would be identifiable in the mass spectra of most ice grains in an Enceladus-like plume.

We identified signatures of an Enceladus model microorganism in both ion modes: positive and negative. While amino acids tend to produce cations under LILBID conditions, fatty acids (potential cell membrane fragments) form deprotonated anions. This strengthens the need for a future impact ionization detector to be capable of detecting both cations and anions (ideally simultaneously) and hence be able to measure the complete range of detectable bacterial cell constituents. By repeatably detecting multiple types of biomolecule components (amino acids and fatty acids), such an instrument would also provide a more robust claim for life detection as emphasized in the Ladder of Life Detection (*57*).

In the cationic mass spectra, we identified molecular peaks and fragments of metabolic intermediates (Fig. 1) as signatures of the bacteria. We found the same amino acids (lysine, arginine, serine, and valine) observed in previous LILBID experiments with extracts of hydrophilic cell compounds of *S. alaskensis* (*48*), together with histidine and threonine. Histidine is a positively charged proteinogenic amino acid that is biosynthesized by most organisms (*58*). Within the cell, it plays an important role in acting as both proton acceptor and proton donor in many enzymatic reactions (*59*). Threonine is a polar proteinogenic amino acid involved in lipid metabolism and protein synthesis (*60*, *61*). Two other metabolic intermediates, nicotinic acid and choline, which play central roles in bacterial metabolism, were also identified (*62*, *63*). Because the laser energy density was chosen to mimic relatively low ice grain impact speeds of 4 to 6 km/s (*41*), elemental ions of masses <18 unified atomic mass unit (u), such as C^+^ or N^+^, were not created in detectable quantities and thus are not expected to be abundant in impact ionization mass spectra of bacterial cells encountered in this speed regime.

The observed fatty acid abundances in our experiments signify the presence of biogenic material and match the fatty acid abundances observed in previous experiments with lipids extracted from *S. alaskensis* (*48*), with peaks of deprotonated heptadecanoic acid (C15:0), hexadecanoic acid (C16:0), and octadecanoic acid (C18:0) showing the highest amplitudes among all identified fatty acids. We labeled peaks in the anionic spectra as unbranched fatty acids (Fig. 2) as they were more abundant in *S. alaskensis* than branched fatty acids (*64*, *65*). However, because these structural isomers have the same molecular mass as unbranched fatty acids, methyl-branched fatty acids (for example, iso and anteiso) might contribute to the lipid pattern observed in the anionic LILBID spectrum. In contrast to Dannenmann *et al.* (*48*), we did not apply any extraction method before conducting the LILBID experiments.

The value of our results and the power of future impact ionization detections is further highlighted by a brief comparison of estimates for the numbers of ions detected in the laboratory and in a high-end flight instrument, such as SUDA (*7*). The total number of ions arriving at SUDA’s multiplier will be in the order of 600,000 to 1,000,000 (~100 to 160 fC) for a typical ice grain, similar to the total number of ions represented in a LILBID spectrum that is co-added from typically 200 to 500 individual spectra (see the “LILBID ion number calculations” section in Materials and Methods). However, the SNR of all signals would be even higher in mass spectra generated by future SUDA-type mass spectrometers considering the higher sensitivity of these instruments (*7*, *10*, *11*) compared to our laboratory mass spectrometer (*41*, *55*). While approximately 100 ions of a single species are sufficient to generate a detectable signal with SUDA, 200 to 700 ions are needed to generate a signal with SNR = 2 using LILBID in the laboratory (see the “LILBID ion number calculations” section in Materials and Methods). The higher sensitivity of spaceborne instruments provides some level of robustness of the idealized experimental conditions that did not account for a mixture of the cell material with non-biogenic organic compounds.

Experiments by Perera and Cockell (*66*) have demonstrated that rapid boiling associated with exposure of fluid to low pressure, a scenario believed to produce droplets from Enceladus’s ocean (*36*), is a potential mechanism for incorporating cells into ice grains in the Enceladus plume. In their experiments, an initial cell density of 6.8 × 10^5^ cells/ml in brine led to the incorporation of a single cell in 1 per 10 (particle diameter < 20 μm) or even 1 per 2 (particle diameter 20 to 100 μm) captured particles, demonstrating that larger grains are more likely to contain cell material than smaller grains. According to these results, assuming a cell density of, for example, only 100 cells/ml in Enceladus’s ocean translates to one cell incorporated in 1 per ~70,000 (diameter < 20 μm) or 1 per ~14,000 (diameter 20 to 100 μm) emitted plume particles. However, ice grains with a diameter of >20 μm are extremely rare in the plume (*39*, *40*, *67*).

Similarly, recent modeling (*68*) demonstrates that sampling one whole cell with a spaceborne instrument that analyzes the average composition of the collected plume material requires more than 100 plume flythroughs, which is more than 0.1 ml of icy material, or a lander with access to a plume surface deposit. Such techniques would measure all possible compounds in the collected material mixed together, making it difficult to separate trace biosignatures from abundant salts and abiotically produced organics. In contrast, our work shows that impact ionization detectors have the unique capability of finding fractions of a cell in a single grain because these instruments are capable of analyzing the compositions of single micrometer-sized ice grains (volume in the order of 1 × 10^−12^ ml; see the “Preparation of *S. alaskensis* cell samples” section in Materials and Methods) emitted by ocean world plumes without sample collection/processing (e.g., preconcentration) or landing.

Impact ionization mass spectrometry takes advantage of the chemical partitioning of different compounds into different ice grains in an ocean world plume, thus providing the assessment of the true compositional diversity of the plume. Integrated analysis cannot capture the strong compositional inhomogeneity nor make use of the fortunate concentration enhancement in single ice grains, potentially diluting astrobiologically relevant organic or inorganic compounds below the limits of detection. Also lost is the ability to correlate composition with ice grain size and spatial distribution in the plume, both of which can provide important insights into ice grain formation and plume eruption mechanics critical for tracing plume compositional measurements back to true composition in the ocean.

Future SUDA-type instruments will be capable of analyzing 10,000 to 100,000 single ice grains during one plume flythrough (depending on altitude and speed). With at least 10 or more flybys during a mission (*8*, *10*, *11*), this would enable the detection of the biosignatures of a fraction of a cell that may be present in just a handful of ice grains among the 100,000s sampled during such a mission. A scenario in which 0.01% of a cell is present in only 1 out of 10,000 or 1 out of 100,000 plume grains resembles a cell density of 2 × 10^3^ or 2 × 10^2^ cells/ml, respectively, if integrated over the entire icy material in the plume (see the “Preparation of *S. alaskensis* cell samples” section in Materials and Methods). The detection of such low cell densities will be hard to achieve by any other analytical method without a lander.

Although an extraterrestrial biosphere might use different biochemistry, it is logical to assume an aqueous-based ecosystem with access to molecular building blocks common in our Solar System [e.g., amino acids, aliphatic hydrocarbons, sugars, nitrogen heterocycles, and others commonly found in meteorites (*69*)] would likely use and modify the concentrations of those molecules in ways that would deviate from an abiotic system (*57*, *70*). Here, we demonstrate the potency of impact ionization mass spectrometry in sampling and identifying several molecular classes of cellular life forms at relevant concentrations in a compositionally heterogeneous plume of an extraterrestrial active ocean world.

## MATERIALS AND METHODS

### Preparation of *S. alaskensis* cell samples

Freeze-dried cultures of *S. alaskensis* (DSM 13593, RB2256) were prepared at the Faculty of Science, Technology, Engineering and Mathematics at The Open University. The cultures were obtained and grown aerobically at 29°C in the Tryptone Soja Broth medium (OxoidTM, Thermo Fisher Scientific). Cells were harvested by centrifugation and the supernatant was discarded. The pellet was resuspended in 0.9% (w/v) aqueous NaCl solution and centrifuged to wash the cell pellet. After discarding the supernatant, the cell pellet was freeze-dried and stored at −20°C until further use.

To prepare the *S. alaskensis* cells for analysis, the freeze-dried cells were transferred into a sterile 50-ml Falcon tube (polypropylene) and resuspended in deionized water for cation measurements or in a mixture of deionized water and isopropanol (1:1 vol %) for anion measurements (cell density of 1 × 10^9^ cells/ml). The *S. alaskensis* cell density in the samples was chosen to simulate the case of one bacterial cell that is incorporated into one ice grain emitted from Enceladus’s plume (cell density calculation see below). Following sonication (37 kHz) of the Falcon tube containing the *S. alaskensis* samples, the samples were then analyzed using the LILBID facility (see the “Simulating mass spectra of single ice grains using the laboratory LILBID facility” section in Materials and Methods).

A single *S. alaskensis* cell has a total volume of <0.1 μm^3^ (*52*). The size of an ice grain from Enceladus is estimated to have a diameter of 1 to 5 μm (*39*, *40*). Assuming a spherical shape, of radius *r*, the volume *V* of an ice grain can be calculated as *V* = 4/3 π*r*^3^. A 2-μm-diameter (1-μm radius) ice grain has a volume of *V* = 4.189 × 10^−18^ m^3^ = 4.189 × 10^−12^ ml. A bacterial cell that is incorporated into one such ice grain results in a cell density of (1 cell)/(4.189 × 10^−12^ ml) or multiplied up to 1 ml: 2.387 × 10^11^ cells/ml.

The dry weight of a cell is estimated to be ~40 fg, half of which is carbon (*71*). This amounts to ~1 × 10^9^ carbon atoms in one cell (carbon weight: 12 u). For comparison, a 70-μm-diameter ice grain (see Discussion) weighs 1.67 × 10^−7^ g (density of 0.917 g/cm^3^) and therefore contains ~5.59 × 10^16^ water molecules (H_2_O weight: 18 u).

With the laboratory LILBID instrument, we simulate single water ice grains by generating single water droplets. These droplets have a diameter of 15 μm (see the “Simulating mass spectra of single ice grains using the laboratory LILBID facility” section in Materials and Methods). Performing the same calculation for a 15-μm-diameter ice grain, the cell density amounts to 0.566 × 10^9^ cells/ml, which constitutes a very low cell density case for an ice grain that is emitted by an ocean world plume. A cell density of 1 × 10^9^ cells/ml was used for the laboratory investigations.

### Simulating mass spectra of single ice grains using the laboratory LILBID facility

The experimental setup used to simulate the mass spectra of ice grains encountered during spacecraft flybys is described in detail in (*41*). A liquid beam containing the prepared *S. alaskensis* samples (see the “Preparation of *S. alaskensis* cell samples” section in Materials and Methods) was injected through a quartz nozzle (diameter of 15 μm) into a vacuum (~10^−4^ mbar). The water beam disintegrates into single droplets after typically 2 to 3 mm (*72*). On average, one droplet contains one bacterial cell (see the “Preparation of *S. alaskensis* cell samples” section in Materials and Methods). A pulsed infrared laser (20 Hz, 7 ns per pulse, 2840-nm wavelength) was focused (~200-μm-diameter laser focus) ~6 mm below the exit of the quartz nozzle into the aforementioned droplet region. When a laser shot hits a droplet (or parts), the liquid sample rapidly disperses into charged and uncharged fragments (*72*, *73*). After passing through a field-free drift region, cations or anions, dependent on the instrument’s polarity, are then accelerated through an electrical field and detected in a TOF mass spectrometer. The mass spectrometer uses the principle of delayed extraction. Setting a predefined delay time between the laser shot and the switch on of the acceleration electrodes allows the extraction of ions as a function of their initial velocities. Combinations of delay times and laser energies can be correlated to ice grain impact speeds onto spaceborne detectors (*41*). The detected mass spectral signals are preamplified and digitized using a LabVIEW-controlled computer.

Depending on the surface area of the liquid droplet irradiated by one laser pulse, mass spectral signals at the same *m/z* value appear at different amplitudes. Spectra resulting from laser pulses that missed the liquid droplets or grazed them were excluded. We only considered those spectra with peaks appearing at notably high amplitudes, meaning laser pulses that hit a particularly large surface area of a single droplet. This selection does not depend on the presence or absence of bacterial cells in the droplets because we only considered peaks that are exclusively related to the matrix solution. The selected spectra, recorded with the same experimental settings, were added and averaged to yield the LILBID spectra shown in this study (Figs. 1 and 2).

Considering the larger laser focus diameter as compared to the droplet size, it is likely that some individual laser shots hit two or more droplets simultaneously. However, the energy distribution within the laser focus diminishes from the center toward the sides, providing the highest ion yield only if the center of the laser focus hits a droplet head-on. Because of the described selection of spectra, most (if not all) events in which two droplets are hit by the same laser pulse should have been excluded and do not contribute to the shown data.

As mentioned above, we used a matrix of deionized water and isopropanol (1:1 volume %) for anion measurements. Isopropanol was needed to help dissolve hydrophobic lipids from the bacterial cells. In contrast to LILBID in the laboratory, cells and lipids in ice grains from space would be incorporated as solids and would not need to be dissolved to be detectable. As demonstrated in earlier LILBID studies with hydrophobic analytes (*46*–*48*), the resulting laboratory spectra still represent a good qualitative and quantitative match with impact ionization mass spectra of ice grains from space. For example, even if 50 vol % of the matrix is an organic solvent, such as isopropanol, water cluster patterns are observable at relative abundances characteristic of ice grain mass spectra from space.

Recorded mass spectra typically have a mass resolution of 600 to 800 m/Dm (full width at half maximum). For one effective measurement, at least 0.3 ml of sample is needed. The experimental setup is calibrated at the beginning of each measurement day using a 10^−6^ M NaCl solution at three different delay time-laser intensity settings. All recorded LILBID spectra are stored in a comprehensive spectral database to aid in planning for future space missions to ocean moons in the Solar System (*55*).

### LILBID ion number calculations

We estimate the absolute number *N* of ions accelerated in the mass spectrometer after being generated by one single laser shot (see the “Simulating mass spectra of single ice grains using the laboratory LILBID facility” section in Materials and Methods) as follows:

$$N=I_{\text{net}}/\left(F_{\text{PA}}*\;e*R_{\text{MCP}}*G_{\text{MCP}}*\;e_{\text{MCP}}\right)$$ (1)

where *I*_net_ is the net integral of the mass range of interest after baseline correction, *F*_PA_ is the amplification factor of the preamplifier, and *e* is the elementary charge (1.6022 × 10^−19^ C). *R*_MCP_ is the impedance of the microchannel plate (MCP) detector. *G*_MCP_ is the gain, that is the multiplication factor of the MCP at an applied voltage. *e*_MCP_ is the detection efficiency that is the probability of an ion being converted into electrons when impacting the MCP detector. The values of *F*_PA_ (10), *R*_MCP_ (50 ohms), and *G*_MCP_ (~5 × 10^5^ to 1 × 10^6^ at the MCP voltages applied here) are given by the manufacturer of the preamplifier and detector (Kaesdorf). *e*_MCP_ of an MCP detector is typically in the order of 0.4 to 0.6 (*74*, *75*).

Using the Origin software (2022b, version 9.9.5.171), we calculate the values of *I*_net_ as follows:

Cation spectrum (Fig. 1): 1.51646 × 10^−7^ Vs.

Anion spectrum (Fig. 2): 1.11584 × 10^−7^ Vs.

Peak of protonated arginine (SNR = 2) in cation spectrum: 4.42165 × 10^−11^ Vs.

Using Eq. 1, and considering the upper and lower values of *G*_MCP_ as well as the range of *e*_MCP_, we calculate an absolute number of 3.155 × 10^3^ to 9.465 × 10^3^ cations, 2.322 × 10^3^ to 6.965 × 10^3^ anions, and 0.920 to 2.760 protonated arginine molecules, respectively, for one single laser shot. Because we co-added 224 and 339 single spectra to achieve the two spectra shown in Figs. 1 and 2, these ion numbers must be multiplied by the number of single spectra to achieve the total number of ions represented in the two spectra:

Cation spectrum: 7.067 × 10^5^ to 2.120 × 10^6^ ions.

Anion spectrum: 7.872 × 10^5^ to 2.361 × 10^6^ ions.

Protonated arginine: 206 to 618 ions.
