# Supplementary material for: How to identify cell material in a single ice grain emitted from Enceladus or Europa
Source: Sci Adv. 2024 Mar 22;10(12):eadl0849. doi: 10.1126/sciadv.adl0849 (PMC10959401; doi:10.1126/sciadv.adl0849)
Supplement: Supplementary file 1 — Figs. S1 to S4 [file sciadv.adl0849_sm.pdf]

Science Advances
AAAS

# Supplementary Materials for

## How to identify cell material in a single ice grain emitted from Enceladus or Europa

Fabian Klenner *et al.*

Corresponding author: Fabian Klenner, fklenner@uw.edu

*Sci. Adv.* **10**, eadl0849 (2024)
DOI: 10.1126/sciadv.adl0849

**This PDF file includes:**

Figs. S1 to S4

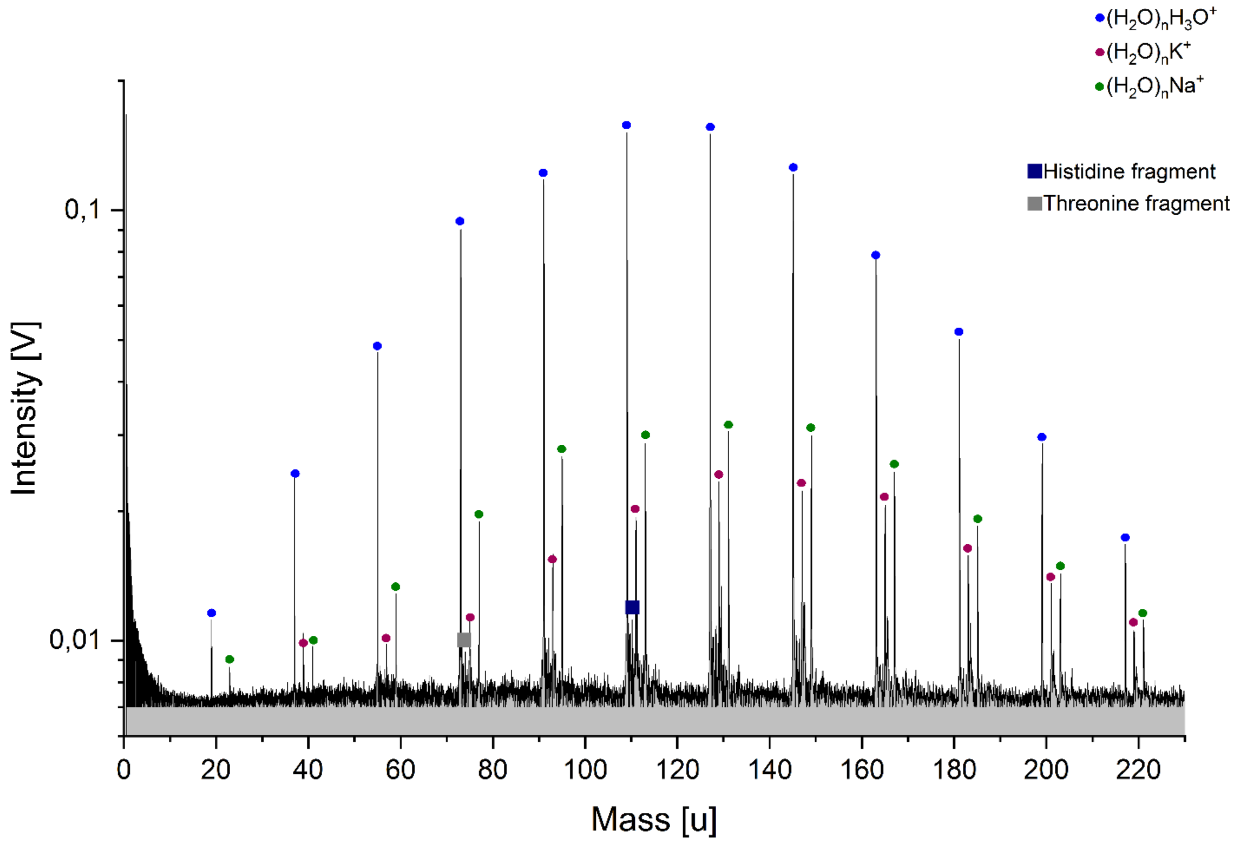


**Fig. S1.**

Baseline corrected cationic mass spectrum of *S. alaskensis* cells at 50 times lower cell densities than needed to simulate the case of a single cell that is embedded in a single ice grain, i.e. only 2 % of one cell in a 15 µm diameter $H_2O$ droplet. Two peaks deriving from the bacterial cell (m/z 74, 110) are still observed (SNR ≈ 2). Compared to the spectrum shown in Fig. 1, the amplitudes of $(H_2O)_nNa^+$ and $(H_2O)_nK^+$ clusters are lower relative to those of $(H_2O)_nH_3O^+$. This agrees with the assumption that these peaks derive mainly from the cell's cytosol. The spectrum is an average of 287 individual spectra recorded with instrument settings corresponding to ice grain impact speeds onto spaceborne detectors of 4-6 km/s (39).

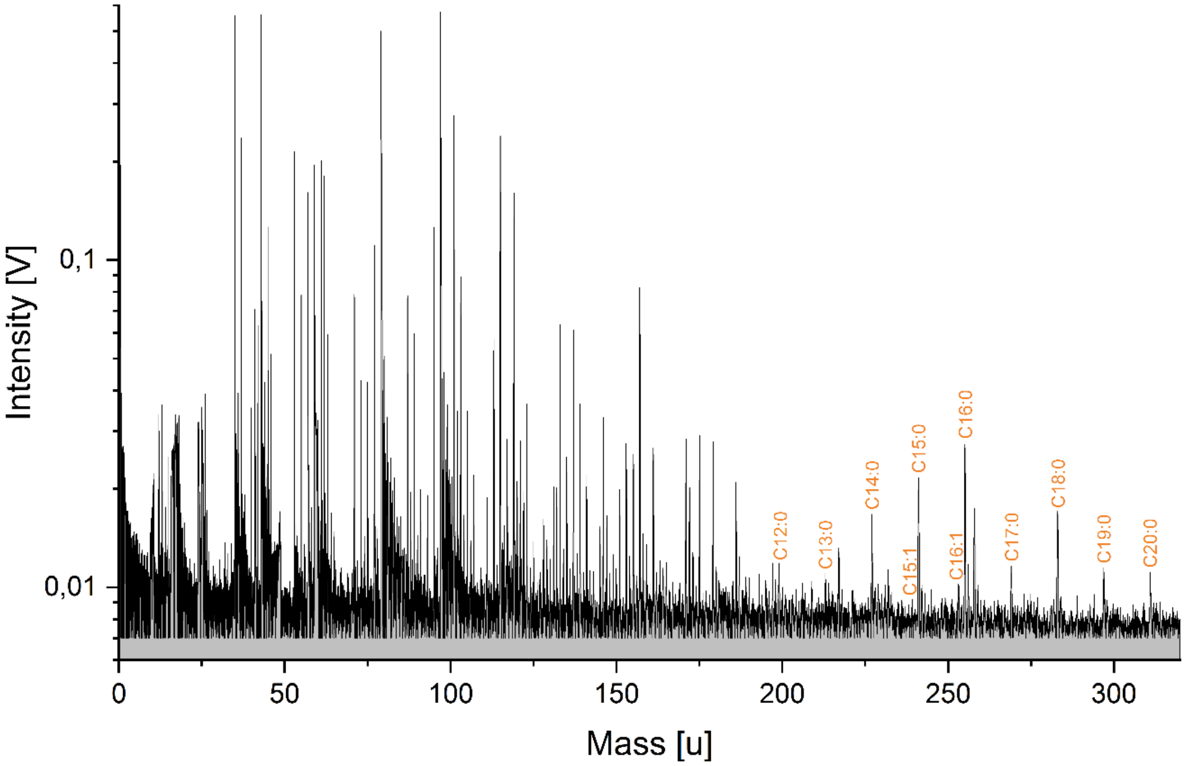


**Fig. S2.**

Baseline corrected anionic mass spectrum of one *S. alaskensis* cell in a 15 µm diameter $H_2O$:isopropanol (1:1 vol:vol) droplet. In addition to fatty acid peaks (as shown in Fig. 2), peaks from the $H_2O$-isopropanol matrix are observed at m/z < 190. The spectrum is an average of 339 individual spectra recorded with instrument settings corresponding to ice grain impact speeds onto spaceborne detectors of 4-6 km/s (39).

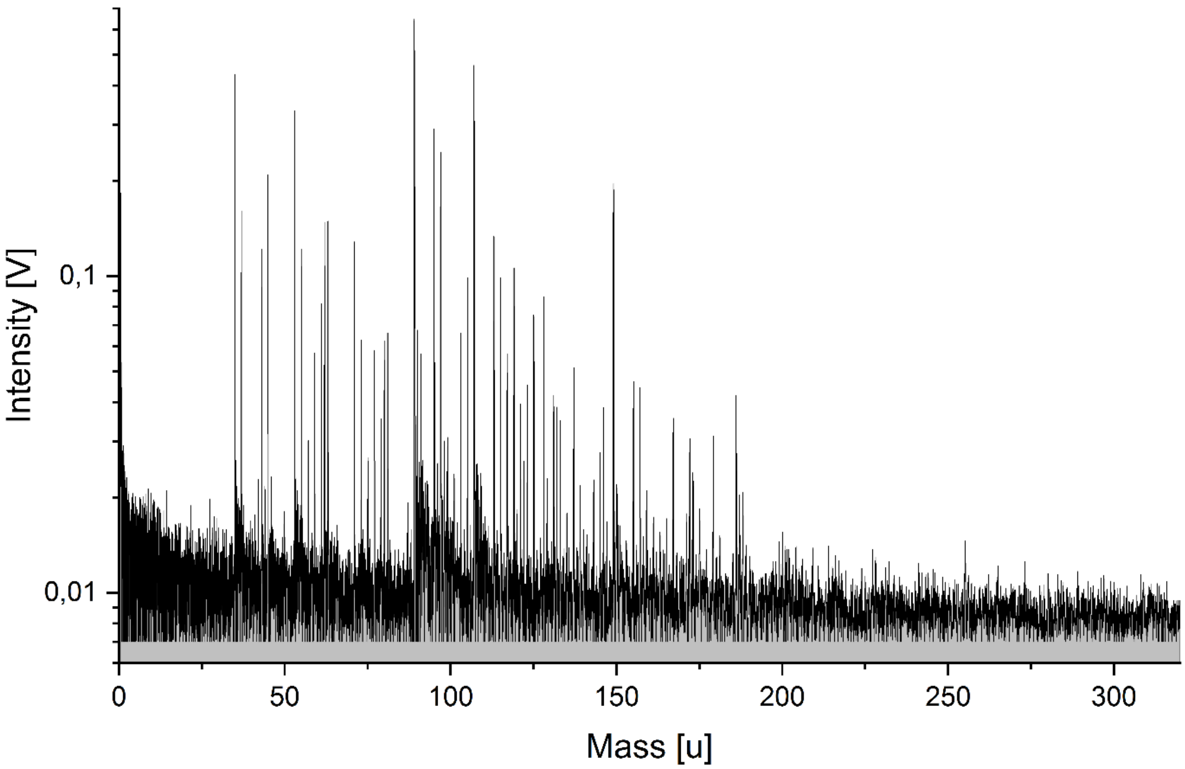


**Fig. S3.**

Baseline corrected anionic mass spectrum of a $H_2O$:isopropanol (1:1 vol:vol) droplet without *S. alaskensis* culture. This spectrum (average of 70 individual spectra) was recorded by using the procedure as described in Materials and Methods 2. No significant peaks are observed at m/z > 190. Instrument settings correspond to ice grain impact speeds onto spaceborne detectors of 4-6 km/s (39).

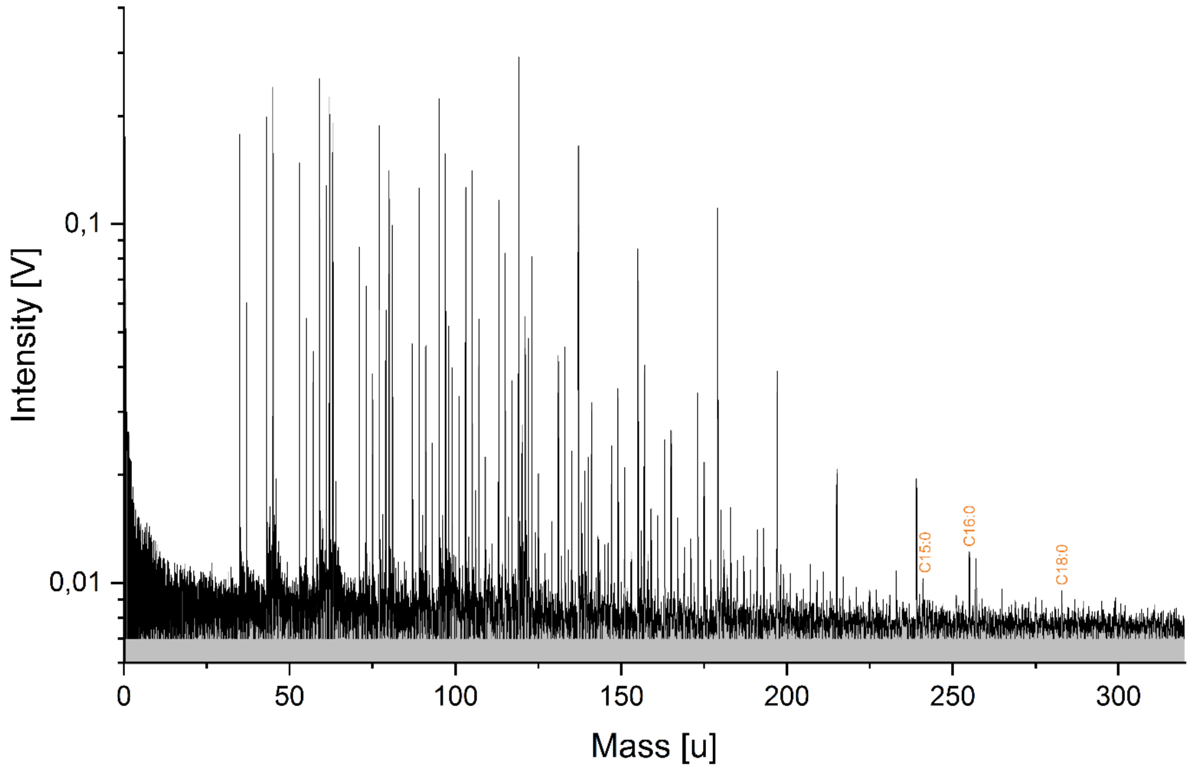


**Fig. S4.**

Baseline corrected anionic mass spectrum of *S. alaskensis* cells at 100 times lower cell densities than needed to simulate the case of one cell that is embedded in a single ice grain, i.e. only 1 % of one cell in a 15 µm diameter $H_2O$:isopropanol (1:1 vol:vol) droplet. In addition to fatty acid peaks at m/z 241, 255 and 283 (C15:0, C16:0 and C18:0), peaks from the $H_2O$-isopropanol matrix are observed. The spectrum is an average of 148 individual spectra recorded with instrument settings corresponding to ice grain impact speeds onto spaceborne detectors of 4-6 km/s (39).
